# Ubiquitin carboxyl-terminal hydrolases are required for period maintenance of the circadian clock at high temperature in Arabidopsis

**DOI:** 10.1038/s41598-019-53229-8

**Published:** 2019-11-19

**Authors:** Ryosuke Hayama, Peizhen Yang, Federico Valverde, Tsuyoshi Mizoguchi, Ikuyo Furutani-Hayama, Richard D. Vierstra, George Coupland

**Affiliations:** 1 Department of Plant Developmental Biology, Max Planck Institute for Plant Breeding Research, Carl-von-Linne Weg 10, D-50829 Cologne, Germany; 2 Department of Natural Sciences, International Christian University, 3-10-2 Osawa, Mitaka, 181-8585 Tokyo, Japan; 3 Department of Genetics, University of Wisconsin-Madison, Madison, Wisconsin 53706 USA; 4 Department of Biology, Washington University in St. Louis, Campus Box 1137, One Brookings Drive, St. Louis, Missouri 63130 USA; 5 Present Address: Bayer Crop Science, 800 N Lindbergh Blvd, St Louis, Missouri 63146 USA; 6 Present Address: Plant Development Unit, Institute for Plant Biochemistry and Photosynthesis, Consejo Superior de Investigaciones Científicas, Universidad de Sevilla, 49th Américo Vespucio Avenue, Sevilla, 41092 Spain

**Keywords:** Gene regulation, Gene expression, Proteases

## Abstract

Protein ubiquitylation participates in a number of essential cellular processes including signal transduction and transcription, often by initiating the degradation of specific substrates through the 26S proteasome. Within the ubiquitin-proteasome system, deubiquitylating enzymes (DUBs) not only help generate and maintain the supply of free ubiquitin monomers, they also directly control functions and activities of specific target proteins by modulating the pool of ubiquitylated species. Ubiquitin carboxyl-terminal hydrolases (UCHs) belong to an enzymatic subclass of DUBs, and are represented by three members in Arabidopsis, UCH1, UCH2 and UCH3. UCH1 and UCH2 influence auxin-dependent developmental pathways in Arabidopsis through their deubiquitylation activities, whereas biological and enzymatic functions of UCH3 remain unclear. Here, we demonstrate that Arabidopsis UCH3 acts to maintain the period of the circadian clock at high temperatures redundantly with UCH1 and UCH2. Whereas single *uch1*, *uch*2 and *uch3* mutants have weak circadian phenotypes, the triple *uch* mutant displays a drastic lengthening of period at high temperatures that is more extreme than the *uch1 uch2* double mutant. UCH3 also possesses a broad deubiquitylation activity against a range of substrates that link ubiquitin via peptide and isopeptide linkages. While the protein target(s) of UCH1-3 are not yet known, we propose that these DUBs act on one or more factors that control period length of the circadian clock through removal of their bound ubiquitin moieties, thus ensuring that the clock oscillates with a proper period even at elevated temperatures.

## Introduction

Selective attachment of ubiquitin is a critical post-translational modification that regulates diverse cellular processes and signaling pathways. Through an ATP-dependent multienzymatic cascade that consists of ubiquitin-activating (E1), ubiquitin-conjugating (E2) and ubiquitin-ligating (E3) enzymes, ubiquitin becomes attached to various proteins through an isopeptide bond between the C-terminal glycine of ubiquitin and typically the ε-amino group of accessible lysines in the target. In some cases, the added ubiquitins become substrates for further ubiquitylation, thus generating concatamers linked internally through any of the seven ubiquitin lysines. While the ubiquitylated protein is often degraded by the 26S proteasome with the release of the ubiquitins for reuse, other non-proteolytic functions also exist where the proteins are reversibly ubiquitylated using de-ubiquitylating enzymes (DUBs) to regenerate the protein in a non-modified form.

DUBs comprise several families of cysteine proteases with specificity for the ubiquitins linked via peptide or isopeptide bonds^1,2^. All DUBs contain a signature catalytic triad of cysteine, histidine and aspartate/asparagine residues and are subclassified as ubiquitin-specific proteases (USPs/UBPs) or as ubiquitin Carboxyl-terminal hydrolases (UCHs) based on the distinctive arrangement of the catalytic site and the presence of other domains^1,2^. Substrates include the initial translation products of ubiquitin genes that often express ubiquitin monomers linked either by a peptide bond to themselves (poly-ubiquitin genes) or to one of two ribosome subunits, and ubiquitylated proteins assembled post-translationally that contain ubiquitins linked via isopeptide bonds either to the target or to other ubiquitins in semi-linear or branch topologies^3^. Other than the global role of DUBs to maintain the pool of free monoubiquitin molecules, some DUBs in mammals have been reported to have more specific functions by associating with the substrate proteins and deubiquitylating them. For example, USP7 and USP10 bind to and deubiquitylate their substrate p53 to mediate its role for suppression of cell propagation upon cellular stresses by counteracting its degradation^4–6^. Similarly, USP42 represents a p53-specific deubiquitylase to play a role in DNA damage-induced p53 stabilization^7^. Also, USP13 interacts to and deubiquitylates receptor-associated protein 80 (RAP80) following DNA damage to activate its ability, promoting DNA-damage responses^8^.

*Arabidopsis thaliana* is predicted to encode a number of DUBs, including at least 16 UBP/USPs and three UCHs, some of which have been confirmed by enzymatic assays^9^. To date, however, few reports have connected individual DUBs to specific substrates. Nevertheless, analyses of mutants that eliminate specific DUBs reveal specificity in their biological functions^9^. For example, in Arabidopsis, the related UBP3 and UBP4 pair has been connected to pollen development^10^, while the UBP12 and UBP13 pair is involved in pathogen immunity, flowering time, and seed development^11–13^. UBP15 and UBP26 have also been linked to flowering and seedling morphogenesis^14,15^, with the latter also important for promoting flowering by reducing transcription of the flowering repressor *FLOWERING LOCUS C*^16^. Within the UCH subfamily, Arabidopsis *uch1* and *uch2* mutants exhibit altered shoot architecture^17^. Notably, these two UCHs impact the turnover of AXR3, a major regulator in the auxin response pathway, implying their direct role for de-ubiquitylation of one or more ubiquitin substrates that modulate auxin signaling^17^.

In this report, we describe a biological function for Arabidopsis UCH3. *UCH3* was originally identified through a mutant screen as a candidate gene necessary for activation of *A. thaliana* CONSTANS (CO), a key transcription factor that promotes flowering under long-day (LD) conditions. This screen was performed in CO overexpressor plants and this role for UCH3 could not be confirmed in wild-type plants, but nevertheless, we found that UCH3, together with UCH1 and UCH2, strongly influences the period of circadian rhythms especially at high temperature. We propose that this set of Arabidopsis DUBs helps maintain the period of the circadian clock at high temperature, thus sustaining appropriate period length at elevated temperatures.

## Results

### Identification and characterization of UCH3 in arabidopsis

*UCH3* was originally identified in a mutant screen as a candidate gene that controls photoperiodic flowering in *Arabidopsis thaliana*. In the Arabidopsis photoperiodic flowering pathway, the B-box type transcription factor CO is activated specifically under LDs to directly induce transcription of *FLOWERING LOCUS T* (*FT*) and promote flowering under these conditions^18,19^. The Arabidopsis line in which *CO* transcription is driven by the constitutive 35S promoter therefore shows a strong early-flowering phenotype with drastically increased *FT* mRNA level^20^. We noticed that this line also shows termination of seedling growth associated with necrosis and chlorophyll bleaching, especially when placed in constant blue light where CO is highly activated (Supplementary Figure 1a)^20^. Growth termination did not appear in WT where the endogenous CO activity is relatively low even if it is grown under blue light (Supplementary Figure 1a). Also, 35S::*CO* could survive in white light where CO is not as strongly activated (data not shown). These results indicate that appearance of the growth-termination phenotype is positively associated with extremely high activity of CO. We therefore mutagenized 35S::*CO* in the *Landsberg erecta* background (Ler), and screened for mutants that exhibit increased survival in continuous blue light and thus are expected to have lower CO activity.

**Figure 1:**
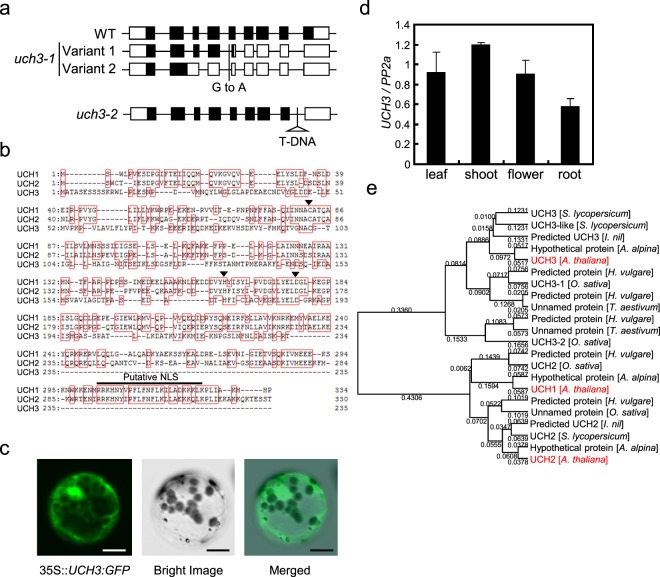
Characterization of the Arabidopsis *UCH3* gene. (**a**) The structure of the genomic region of *UCH3* locus and predicted gene structure in *uch3-1* and *uch3-2* mutants. Squares and lines indicate exons and introns within the gene, respectively, with the gene structure of *uch3-1* specifically obtained from sequencing of *uch3* mRNA species in this mutant. Black squares indicate putative protein-coding regions, with those in the *uch3-1* mutant estimated from sequences of the two transcribed mRNA variants. The position of the point mutation (G to A) in *uch3-1* and that of the T-DNA in *uch3-2* within the *UCH3* loci were also indicated in the gene region. (**b**) Comparison of the deduced amino-acid sequence between UCH1, UCH2 and UCH3. Red squares indicate amino-acid residues identical among at least two represented UCHs. Arrows indicate putative amino-acid residues that comprise the catalytic triad conserved among Arabidopsis UCHs. Putative NLS present in the deduced amino-acid sequences of UCH1 and UCH2 were shown by a line. (**c**) Intracellular localization of UCH3. The plasmid that carries 35 S::*UCH3:GFP* was transfected into *A. thaliana* protoplasts to check intracellular localization of UCH3:GFP. Scale bars indicate 10 μm. (**d**) Expression levels of *UCH3* at Arabidopsis tissues. *UCH3* expression was examined in *A. thaliana* Col leaves, shoots, flowers and roots. Error bars indicate SE of two biological replicates. (**e**) Phylogenetic relation among the whole UCH-related proteins in seven higher plants with uncovered whole genomic sequences. The phylogenetic tree was constructed by the UPGMA method.

Subsequent positional cloning of one of the four isolated mutants identified a mutation in the *UCH3* gene locus. This mutant, *uch3-1*, significantly reduced the level of *FT* mRNA under LD condition with 16 h light/8 h dark, as well as showing increased survival under constant blue light (Supplementary figure 1a,b), consistent with the idea that *UCH3* participates in the control of CO activity. The mutant had a G-A substitution at the boundary of the 4^th^ intron and the 5^th^ exon, which led to two abnormally spliced mRNA variants predicted to produce truncated UCH3 proteins (Fig. 1a, Supplementary Fig. 1g). In mRNA variant 1, the 5′ region of the 5^th^ exon was spliced to the 4^th^ intron, which led to fusion of the 4^th^ exon and the rest of the 5^th^ exon to generate a premature stop codon (Fig. 1a). Variant 2 lacked the same exon region, but also had a defect in splicing of the 2^nd^ intron, which caused fusion of the 2^nd^ exon, 2^nd^ intron and 3^rd^ exon to produce a long single exon region and a premature stop codon (Fig. 1a).

Analysis of the full sequence of UCH3 revealed that it encodes a DUB related to the previously characterized hydrolases UCH1 and UCH2 with 46 and 47% amino acid sequence similarity, respectively^17^. The three sequences were contiguous across most of the polypeptides, including the cysteine, histidine and aspartic acid residues that comprise the catalytic triad, except for UCH3 which was missing 50 residues at the C-terminal end (Fig. 1b). This region harbors a predicted nuclear localization sequence, which may be related to UCH1 and UCH2 localization to the nucleus^17^. To test intracellular localization of UCH3, GFP-fused UCH3 was constitutively expressed from the 35 S promoter in *A. thaliana* protoplasts. In this experiment, UCH3:GFP accumulated in the cytoplasm as well as in the nucleus (Fig. 1c), indicating that it shares the same intracellular location with UCH1 and 2. We also examined in which tissues *UCH3* is expressed. We tested expression of this gene in leaves, shoots, flowers and roots in *A. thaliana*, and found that it is expressed similarly in each of these tissues (Fig. 1d), suggesting that *UCH3* is broadly expressed within Arabidopsis.

Predicted protein coding sequences related to Arabidopsis UCHs were detected in the genomic sequences of seven higher plants (*A. thaliana*, *Arabis alpina*, *Solanum lycopersicu*m, *Ipomoea nil*, *Oryza sativa*, *Hordeum vulgare*, and *Triticum aestivum*). This analysis showed that they are clustered into five subgroups in the phylogenetic tree, with Arabidopsis UCH1, UCH2 and UCH3 separately belonging to three distinct clusters (Fig. 1e). Although Arabidopsis UCH1 and UCH2 were clustered into two separated subgroups with the evolutionarily related proteins of both monocot and dicot, Arabidopsis UCH3 was clustered with proteins of dicots alone, with UCH3-related proteins of monocots independently being populated in the phylogenetic tree forming a single subgroup. Also, an additional monocot-specific type of UCH, which is relatively similar to UCH3 at the level of the amino-acid sequence, was represented as a single independent cluster in the phylogenetic tree (Fig. 1e).

### UCH3 possesses broad deubiquitylation activities

**Figure 2:**
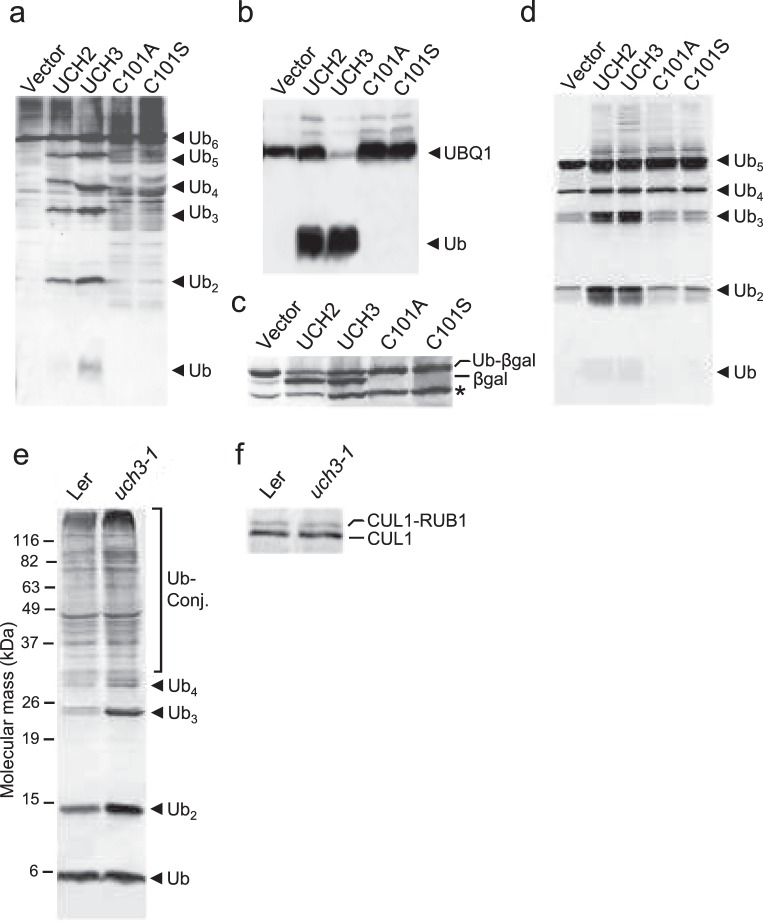
UCH3 possesses broad deubiquitylation activities. (**a**,**b**) Deubiquitylation activities of UCH3 against the Arabidopsis ubiquitin hexameric protein UBQ10 and an Arabidopsis ubiquitin extension protein UBQ1. UCH2, UCH3, and its mutant variants (C101A and C101S) were co-expressed with UBQ10 (**a**) or UBQ1 (**b**) in *E. coli*, and the cleavage products (Ub_5_, Ub_4_, Ub_3_, Ub_2_ and Ub for UBQ10, and Ub for UBQ1), together with UBQ10 and UBQ1 themselves, were detected by using an anti-ubiquitin antibody. (**c**) The deubiquitylation activity of UCH3 against the ubiquitin-βgal fusion protein. ubiquitin-βgal was co-expressed in *E.coli* and cleavage of βgal was detected by an anti-βgal antibody. The asterisk indicates the ω-fragment of β-gal constitutively expressed in the *E. coli* Nova Blue (DE3) strain. (**d**) *in vitro* cleavage of Lys48-linked poly-ubiquitin chains by UCH3. (**e**) Endogenous levels of polyubiquitylated proteins in the *uch3-1* mutant. The levels of total ubiquitin conjugates and free poly-ubiquitin chains was analyzed by immunoblot analysis with an anti-ubiquitin antibody. (**f**) Effects of the *uch3-1* mutation on accumulation of the CUL1-RUB1 conjugate and CUL1. The level of these proteins were analyzed by immunoblot analysis with an anti-CUL1 antibody.

The enzymic activity of UCH3 had not previously been tested, and therefore recombinant UCH3 was expressed in *E. coli* and tested for de-ubiquitylation activity. In this assay, UCH3 was found to have broad specificity against a range of possible substrates. As compared to a similarly promiscuous paralog UCH2, UCH3 was effective in releasing ubiquitin moieties concatentated through peptide linkages, using as substrates either a hexameric polyubiquitin expressed by the *A. thaliana UBQ4* locus, or a ubiquitin-ribosome fusion from the *A. thaliana UBQ1* locus (Fig. 2a,b). Notably, substitution of the active site cysteine for either an alanine (C101A) or serine (C101S) blocked this activity. Using a Ub-β-galactosidase fusion with the ubiquitin moiety fused to β-galactosidase polypeptide through a methionine amino acid, we also found that UCH3 successfully cleaves this substrate leading to the release of β-galactosidase (Fig. 2c). To test for cleavage of isopeptides linkages, we incubated recombinant UCH3s with purified chains of ubiquitin linked internally through Lys-48. As described above for other substrates, wild-type UCH3 but not its C101-A or C101-S variants processively disassembled these chains *in vitro* with the concomitant appearance of free ubiquitin (Fig. 2d).

The loss of UCH3 might be expected to impact on endogenous levels of ubiquitylated proteins, so the levels of total ubiquitin conjugates were examined by immunoblot analysis of total seedling extracts. Alhough the overall levels of ubiquitin conjugates were not strongly changed in the *uch3-1* mutant seedlings, free poly-ubiquitin chains were clearly elevated as compared to the Ler wild type (Fig. 2e). On the other hand, no significant change in the level of monoubiquitin was observed (Fig. 2e). Given the possibility that UCH3 could disassemble conjugates containing other members of the ubiquitin-fold family, we tested the impact of the *uch3-1* mutant on levels of Cullin1 modified with Rub1, the closest member of the ubiquitin family to ubiquitin. No effect on the *in vivo* level of the Cullin1-Rub1 conjugate was seen (Fig. 2f). Taken together, it appears that UCH3, like its UCH2 relative, will release ubiquitins from an array of peptide- or isopeptide-linked substrates, and will specifically influence the profile of ubiquitylated species seen *in planta*.

### UCH3 acts redundantly with UCH1 and UCH2 to influence flowering time of Arabidopsis independent of CO activation

**Figure 3:**
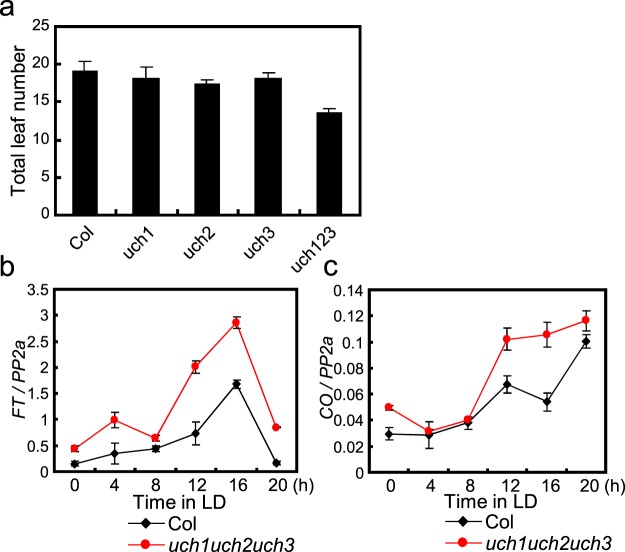
The *uch1uch2uch3* mutant exhibits early flowering with elevated expression of photoperiodic-flowering genes. (**a**) Flowering time of *uch1*, *uch2*, *uch3*, and *uch1 uch2 uch3* triple mutants under LDs. Plants were grown under 16 h light/ 8 h dark, and the number of total leaves was counted at the flowering stage. Error bars indicate SD among 16 plants. (**b**,**c**) Expression pattern of *FT* (**b**) and *CO* (**c**) under LD. 12-day-old plants grown under 16 h light/ 8 h dark were collected every 4 h over 24 h to perform expression analysis. *PP2a* was used for the control. Error bars indicate SE among two biological replicates.

The fact that levels of *FT* mRNA were reduced in the isolated *uch3-1* mutant in 35S::*CO* background suggested that they might also be lower in *uch3-1* in wild-type plants. Unexpectedly, a significant change in *FT* mRNA expression was not observed in this mutant line (Supplementary Fig. 1c). We also obtained another Arabidopsis mutant line in which a T-DNA was inserted into the *UCH3* gene locus (*uch3-2*; SALK-023266). In this line, with a T-DNA being inserted into the 8^th^ intron within the *UCH3* gene region (Fig. 1a), transcripts of *UCH3* were not detected, indicating that the inserted T-DNA severely affects mRNA stability of *UCH3* (Data not shown). We crossed this line to 35S::*CO* to create *uch3-2*/35S::*CO*, and checked flowering time and *FT* expression. We could confirm that *uch3-2*/35S::*CO* exhibits increased survival in blue light with significantly reduced levels of *FT* mRNA under LD (Supplementary fig. 1d,e), whereas like *uch3-1*, the original *uch3-2* mutant did not exhibit a reduction in *FT* accumulation (Supplementary Fig. 1f). As these results suggest that *UCH3* might act to induce *FT* expression redundantly with *UCH1* and *UCH2*, we crossed *uch3-2* to Arabidopsis T-DNA inserted mutant lines for these genes to obtain and analyze the triple *uch1uch2uch3* mutant. Contrary to our expectations, this line exhibited slightly early flowering under LDs (Fig. 3a). Consistently, levels of *FT* expression, as well as those of *CO*, were also elevated compared to WT under LD (Fig. 3b,c). These results suggest that reduced *FT* expression previously observed in *uch3* mutants with 35S::*CO* was caused by an effect of the *uch3* mutation against an artificially elevated CO activity through overexpression of *CO* from the 35S promoter, and that *uch3* mutations do not reduce CO activity at wild-type levels of expression. On the other hand, the *uch3* mutation, together with *uch1* and *uch2*, clearly increases transcript levels of *CO* and *FT* in the WT background, indicating that UCH1/2/3 represent a set of DUBs that impact flowering by decreasing expression of *CO* and *FT* transcripts in Arabidopsis.

### UCH3 acts with UCH1 and UCH2 to maintain the period of the circadian clock especially at high temperatures

In the Arabidopsis photoperiodic flowering pathway, the circadian clock controls transcription of *CO* to generate its particular diurnal pattern^21^. Since the daily pattern of *CO* was significantly affected in the triple *uch1 uch2 uch3-2* mutant, we tested whether the function of the circadian clock is altered in *uch* mutants.

In *A. thaliana*, the circadian oscillator consists of multiple interlocked transcriptional feedback loops containing several transcriptional repressors^22–24^. A pair of Myb-related transcription factors LATE ELONGATED HYPOCOTYL (LHY) and CIRCADIAN CLOCK ASSOCIATED 1 (CCA1), together with another transcription factor TIMING OF CAB EXPRESSION 1 (TOC1) form a transcriptional loop in the circadian clock, so that LHY/CCA1 directly repress transcription of *TOC1* and TOC1 in turn suppresses transcription of *LHY/CCA1*^25–27^. TOC1 is a member of the PSEUDO RESPONSE REGULATOR (PRR) family that also contains PRR9, PRR7 and PRR5, which are sequentially expressed during the day to also suppress transcription of *LHY*/*CCA1*^28–30^. In addition to TOC1, LHY/CCA1 also directly repress transcription of *PRR9*, *PRR7* and *PRR5*, forming another layer of the transcriptional feedback loop^31^. Reciprocal transcriptional suppression is also observed between LHY/CCA1 and the protein complex called the evening complex (EC), which consists of evening-expressed Myb-related transcription factor LUX ARRYTHMO (LUX) and two nuclear proteins, EARLY FLOWERING 3 (ELF3) and EARLY FLOWERING 4 (ELF4)^31–33^.

**Figure 4:**
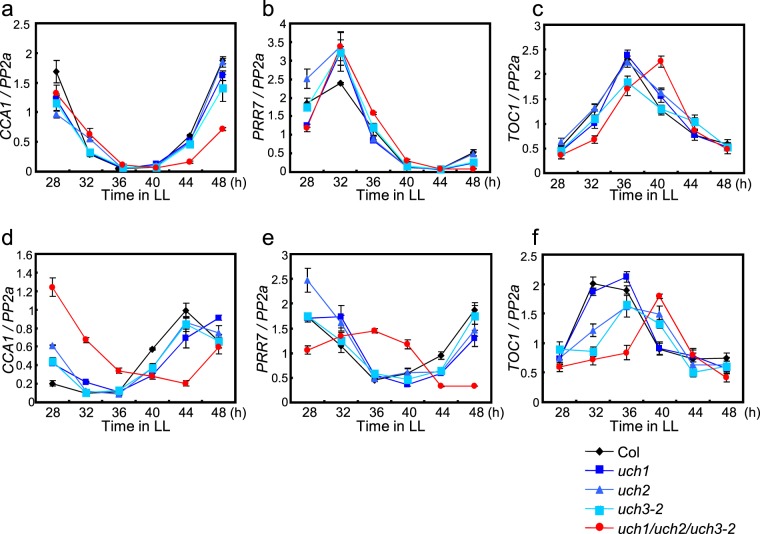
*UCH1*, *UCH2* and *UCH3* together affect circadian rhythmicity in gene expression at a high temperature. Plants entrained to 12 h light/12 h dark at 22 °C for 12 days were sifted to LL at 22 °C (**a**–**c**) or 29 °C (**d**–**f**). Expression of *CCA1* (**a**,**d**), *PRR7* (b and e), and *TOC1* (**c**,**f**) was measured every 4 h between 28 h and 48 h after transfer to LL. Error bars indicate SE of two biological replicates.

We first examined the effect of individual *uch1*, *uch2* and *uch3* mutations and *uch1 uch2 uch3* triple mutations on rhythmicity in expression of several Arabidopsis clock genes. We entrained WT, single *uch1*, *uch2*, *uch3* and triple *uch1 uch2 uch3* mutants to 12 h light/12 h dark, transferred these lines to continuous light (LL) and examined *CCA1*, *PRR7* and *TOC1* expression every 4 h over 24 h between time 28 and 48 after transfer to LL at 22 °C (Fig. 4). *uch1*, *uch2* and *uch3* single mutants did not show significant changes in the rhythmic expression of these genes. However, the triple *uch1 uch2 uch3* mutant exhibited slight delays in the phase of expression of these genes during the range of time tested (Fig. 4a–c). Interestingly, changes in the phase of gene expression were clearly enhanced when plants were transferred in LL at a higher temperature. At 29 °C, single *uch1*, *uch2 and uch3* mutants exhibited weak but significant changes in the phase of *CCA1*, *PRR7* and *TOC1* rhythms compared to those at 22 °C (Fig. 4d–f, Supplementary Fig. 2). Moreover, the triple mutant exhibited clearer phase delays in circadian expression of these genes compared to the normal temperature (Fig. 4d–f). The *uch3-1* mutant originally identified through the mutant screen also showed a similar clock phenotype at 29 °C, indicating that UCH3 isolated through this screen indeed influences clock function (Supplementary figure 1 g). These results indicate that UCH1, UCH2 and UCH3 additively affect circadian rhythms of clock-gene expression at high temperature.

**Figure 5:**
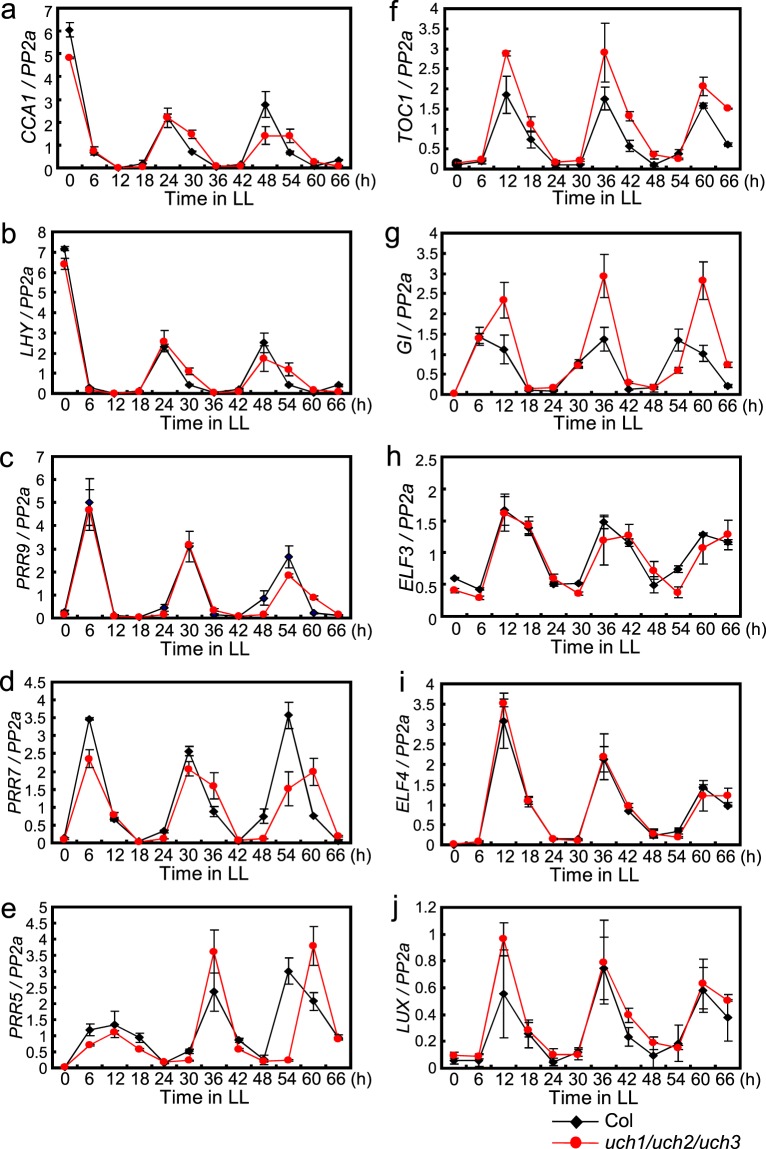
The effect of *uch1 uch2 uch3* mutations on circadian expression of a series of clock genes at 22 °C. Plants entrained to 12 h light/12 h dark at 22 °C for 12 days were sifted to LL at the identical temperature. Expression rhythms of a series of clock genes were monitored every 6 h over 66 h in LL. Error bars indicate SE of two biological replicates.

**Figure 6:**
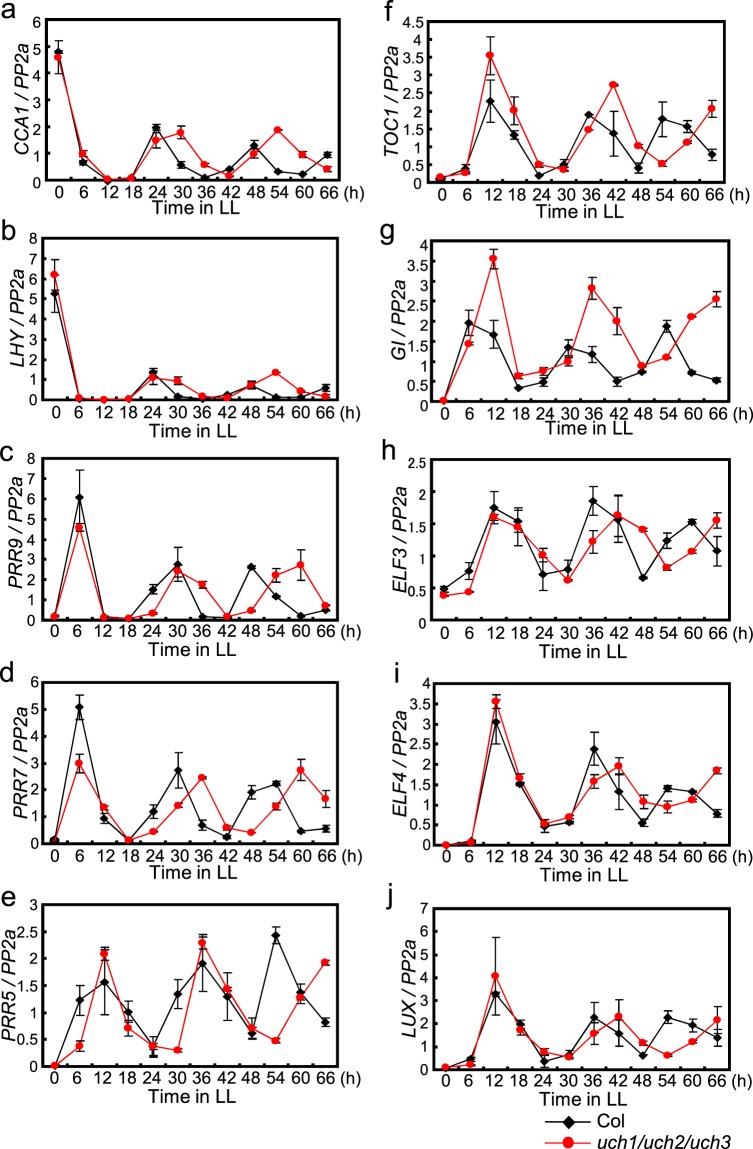
The effect of *uch1 uch2 uch3* mutations on circadian expression of a series of clock genes at high temperature. Plants entrained to 12 h light/12 h dark at 22 °C for 12 days were sifted to LL at 29 °C. Expression rhythms of clock genes shown in Fig. 5 were monitored every 6 h over 66 h in LL. This experiment was carried out at the same time with that represented in Fig. 5. Error bars indicate SE of two biological replicates.

To understand more thoroughly the effect of the *UCH* genes on the regulation of circadian rhythms at high temperatures, we examined in *uch* triple mutants the rhythmic expression of Arabidopsis major clock genes in LL at 29 °C (Figs. 5, 6). We entrained WT and the *uch1 uch2 uch3* mutants to 12 h light/12 h dark and transferred to LL at 22 °C and 29 °C to check gene expression every 6 h over 66 h in LL. At 22 °C the triple mutants caused only slight period lengthening of circadian expression of a series of Arabidopsis clock genes (Fig. 5), together with increased amplitude of the *GI* rhythm, respectively (Fig. 5g). However, at 29 °C the triple mutants more drastically lengthened the period of circadian expression of these genes, progressively delaying the phase of expression in later cycles under LL (Fig. 6). Elevated amplitude in the expression rhythm of *GI* was also maintained at 29 °C (Fig. 6g). To more clearly examine the function of UCH3 on the period of the circadian clock at high temperature, we compared circadian expression of several clock genes in *uch1 uch2 uch3* triple mutants to those in the *uch1 uch2* double mutants. Increased period length of both *LHY* and *GI* expression rhythms in LL at high temperature were observed in the *uch* triple mutant compared to the double *uch* mutant, confirming the function of UCH3 to maintain the period of the circadian clock at high temperature (Supplementary fig. 3a). Together, these results indicate that UCH3, as well as UCH1 and UCH2, influence the period of the circadian clock specifically at high temperatures in Arabidopsis. To check the influence of the period lengthening phenotype in the *uch* triple mutant within light/dark (LD) cycles, we tested diurnal expression of several Arabidopsis clock genes in 12 h light/12 h dark at 29 °C. The phase of *LHY*, *CCA1* and *GI* rhythms was not affected in *uch1 uch2 uch3* mutants, although increased amplitude in the diurnal pattern of *GI* expression was observed in this mutant (Supplementary fig. 3b). We also examined the expression pattern of *UCH3* in LL at 29 °C to determine whether its expression exhibits a circadian rhythm. *UCH3* mRNA level was broadly constant in LL, indicating that the level of *UCH3* transcripts is not controlled by the circadian clock (Supplementary Fig. 3c).

## Discussion

In this study, we characterized the function of the *UCH3* gene in *Arabidopsis thaliana*, as well as reporting a novel function of previously functionally characterized *UCH1* and *UCH2*. Though the single *uch3* mutation did not strongly alter circadian expression of Arabidopsis clock genes, combining it with *uch1* and *uch2* strongly affected circadian expression specifically at high temperature. We propose that Arabidopsis UCHs are involved overall in maintenance of circadian clock functions especially at high temperatures, ensuring that the clock oscillates with a proper period at elevated temperatures.

Through both *in vivo* and *in vitro* assays, we confirmed that UCH3 has broad activities to generate free ubiquitins by acting on substrates that link ubiquitin via peptide and isopeptide linkages. Despite its potential universal role for ubiquitin cycling, *uch3* mutants were viable and complete the entire life cycle with no obvious effects on their morphology. Therefore, the effect of *uch3* mutations on overall dynamics of ubiquitin cycling may be limited, perhaps due to redundancy with other DUBs. In general, despite global roles of DUBs for ubiquitin cycling, mutations in genes that encode DUBs often cause phenotypic changes in specific physiological processes in Arabidopsis. For example, *ubp13* and *ubp14* single mutants both exhibit early flowering under short days (SDs) with significantly altered expression of several photoperiodic-flowering genes^12^. Also, the *ubp15* mutant displays changes in leaf development^14^. Tissue specific expression might contribute to generating specificity in their physiological functions. Consistent with this idea, expression of *UBP15*, with its disruption causing altered leaf morphology, mainly occurs in leaves^14^. However, tissue specific expression of DUBs does not entirely explain specificity in their biological functions. For example, UCH1 and UCH2 have been reported to be ubiquitously expressed in Arabidopsis, whereas the impact of loss of these functions is limited to particular physiological processes such as development of shoot architecture. Rather, specificity in their biological functions is likely to be caused by their selectivity against poly-ubiquitylated proteins. Consistently, Arabidopsis UBP12 has been recently reported to deubiquitylate the ubiquitin monomer linked to histone 2A^34^. In animal, USP7, USP10 and USP42 bind to and deubiquitylate the substrate p53 to counteract its degradation^4–7^. Also, USP13 interacts with and deubiquitylates RAP80 to activate its function^8^.

Our study demonstrates a specific biological function for Arabidopsis UCHs. We showed that *uch1 uch2 uch3* triple mutants exhibit slightly early flowering, increased *FT* expression and altered diurnal pattern of *CO* transcription under LD, with also slightly altered circadian rhythms of clock-gene expression in LL at 22 °C. The effect of the triple *uch* mutations on circadian expression of a series of Arabidopsis clock genes in LL was more apparent at high temperature, indicating that UCHs possess the role in maintaining the period of the circadian clock specifically at high temperatures. The effect of *uch* mutations on the period of the circadian clock specifically at high temperature suggests possible roles in mediating temperature compensation, which allows the clock system to maintain constant period length across a wide range of temperatures. However, since the temperature compensation mechanism is predicted to lengthen the clock period at high temperatures to counteract its period being shortened with rising temperature^35^, UCHs, which function to shorten the period of the clock at high temperatures, may not be factors that directly provide temperature compensation of the clock at high temperatures. However, an alternative possibility is that UCHs fine-tune the temperature compensation mechanism by reducing its activity of lengthening the period of the clock at high temperature to avoid the mechanism “over-compensating” for the period changes. In this scenario, the temperature compensation mechanism can precisely adjust the activity of the clock system at high temperatures. The specific role of UCHs might be to mediate this response by influencing *GI* expression, since triple *uch* mutations not only altered the period of this gene but also significantly increased its amplitude in LL. A previous report has shown that *GI* is crucial for circadian rhythmicity at high temperatures; although the *gi* mutation does not influence circadian rhythms of clock genes such as *LHY*, *CCA1* and *TOC1* at normal growth temperatures, it strongly disrupts their circadian rhythms at higher temperatures^36^. On the other hand, temperature compensation of the circadian clock in Arabidopsis is also mediated by other multiple mechanisms that control distinct transcriptional and post-translational processes in the core of the clock^37,38^, so UCHs can also influence temperature compensation of the clock by impacting specific factors within these mechanisms. For example, disruption of *HEAT SHOCK FACTOR B2b* (*Hsf2b*), that encodes a direct transcriptional repressor of *PSEUDO RESPONSE REGULATOR 7* (*PRR7*), shortens the circadian period specifically at high temperature^39^. Also, CKB4, a regulatory subunit of the casein kinase 2 (CK2) that physically interacts with CCA1 to mediate its phosphorylation, was reported to confer temperature compensation in the clock by counteracting high-temperature mediated increases in affinity of CCA1 for its target clock genes^40^. Moreover, FLOWERING BASIC HELIX-LOOP-HELIX 1 (FBH1), a transcription factor that binds to the promoter of *CCA1* to control its transcription, has also been identified as a component of temperature compensation of the clock in Arabidopsis^41^. UCHs might affect temperature compensation of the clock by modulating the functions of specific factors within these mechanisms that include Hsf2b, CKB4 and FBH1 through their deubiquitylation activities to remove bound ubiquitin moieties from target proteins in Arabidopsis.

In this report we investigated a novel UCH function to maintain the period of the circadian clock at high temperature. However, how UCHs mediate temperature compensation of the Arabidopsis circadian clock is still unknown. UCH1 and UCH2 have been previously reported to be involved in the control of shoot architecture by impacting turnover of a representative AUX/IAA protein AXR3 within the auxin signaling pathway^17^. As previously described, turnover of other unrelated proteins such as PHYTOCHROME A (PHYA) and LONG HYPOCOTYL 5 (HY5), far-red light photoreceptor and a transcription factor in the light signalling pathway, respectively, does not change in the double mutant^17^, implying that UCHs possess specific protein targets. Further molecular analyses such as discovering proteins that are directly associated with and modulated by UCHs through their deubiquitylation activities, may lead to progress in our understanding of how UCHs control temperature compensation of the circadian clock in Arabidopsis.

## Materials and Methods

### Plant materials and growth conditions

Direct mutagenesis of 35S::*CO* (the Landsberg background) was performed by treating approximately 30,000 seeds with ethylmethane sulfonate (EMS). The obtained M1 seeds were separately sown on soil to obtain 250 pools of M2 seeds. A portion of M2 seeds from each of these pools were used for screening for mutants. For the screening, the M2 seeds sown on agar plates were placed in a growth chamber with continuous blue light that induces the activity of CO, and plants that survived under this condition were selected. The obtained *uch3-1* mutant was back-crossed with Landsberg four times to remove unrelated mutations within the genome. *uch3-2* was obtained as the SALK T-DNA insertion line (SALK-023266), backcrossed with Columbia (Col) four times for removing unrelated mutations. *uch1* and *uch2*, T-DNA insertion mutants in the Wassiliewskija (Ws) ecotype, were originally characterized by Yang *et al*. For creating *uch1*, *uch2*, and triple *uch1uch2uch3* mutants in the Columbia background, the *uch1uch2* double mutant in Ws was backcrossed with Col four times to first obtain *uch1uh2* in Col. The double mutant was then crossed with the *uch3*-*2* mutant to obtain the *uch1uch2uch3* triple mutant in Col. *uch1* and *uch2* single mutants in Col were obtained as F2 segregants after the cross between *uch1uch2* in Col and *uch3-2*.

### Deubiquitylating enzyme activity assays

The ability to release Ub linked via α-amino linkages was assayed *in vivo* using the substrates: ubiquitin-extension protein *At*UBQ1 (p8185) and the hexameric polyubiquitin *At*UBQ10^3,42^; the latter had the transcription start site modified to attenuate expression (*p*At*UBQ10-LE* as described in Yan *et al*.^43^. The coding regions for each substrate was introduced into a pACYC184-based plasmid and was co-expressed in the *E. coli* strain NovaBlue (DE3) (Novagen) with wild-type *UCH3* or its C101A or C101S mutant version present in pET32a. The Ub-Met-β-galactosidase substrate was as described by Papa and Hochstrasser^44^. Similarly expressed UCH2 was included as a positive control.

The cleavage of Ubs connected via ε-amino isopeptide bonds used lysine-48-linked poly-Ub chains synthesized *in vitro* by the wheat E2 UBC7 as described^45^. Extracts containing the recombinant enzymes were prepared by concentrating cells expressing the corresponding proteins in 1/20 of the original culture volume and sonicating the cells in 50 mM Tris-HCl (pH 8.0), 5% (v/v) glycerol, 1 mM dithiothreitol, and 1 mM Na_4_EDTA. Lysates (37.5 µl) clarified at 10,000 × *g* were incubated for 2 hr at 37 °C with 2.5 µl (50 ng) of poly-Ub chains. The reactions were quenched by heating after the addition of SDS-PAGE sample buffer. The processing of the various substrates for UCH3 was monitored by SDS-PAGE and immunoblot analysis with anti-Ub or anti-B-galactosidase antibodies^43^.

Ubiquitin conjugates and the Cullin-Rub1 adduct synthesized *in vivo* were assayed by SDS-PAGE and immunoblot analysis of total seedling extracts with anti-Ub and anti-Cullin1 antibodies^45^.

### RNA expression analyses

RNA was isolated by RNasy Plant Mini Kit (Qiagen). cDNA synthesis and removal of genomic DNA were performed with PrimeScript™ RT reagent Kit with gDNA Eraser (TaKaRa). Expression analyses were performed by real-time PCR (AriaMx Real-Time PCR system in Agilent Technology). Primers used for the expression analyses are; *CO* (GCATGTGTCACAACAGCTTCAC and ATGCCTTCCTCGAAGCATACC), *FT* (TCAGAGGGAGAGTGGCTG and TCACCGTTCGTTACTCGTATC), CCA1 (CATGTGGAACAGAAAGATCCCAAA and TGTTACAGGAAGACTATGGACAAGG), LHY (AGTCTGCAAAAGGCTTCGATTG and TGGTACAGAACCTGACATGACC), *PRR9* (CCTCGAGTGAAAGGCCAGT and CAAAAGTTGCCCCAGTATCTCA), PRR7 (CGAGGCCAATTTGTGCGT and TTGGGCTGAGAAATAGTGGGTT), *PRR5* (CGTTCGTCAAGTCCAATCCAC and AGAACAGCTCCTGCATCGG), *TOC1* (CTGCTGACTATGATGACGAGGA and AAGAGCCAACATTGCCTTAGAG), *GI* (ATGCCTACTCAGTTTCTCGACA and CGAGCGAGAGCAAATCCAAC), *ELF3* (GACTCGGAGAAGACTGACCAA and GGTTGTTTGCAAAAGGCATGT), *ELF4* (ACGGAAACATTTCCAAGGTTGT and CCGGTTCATTAAGCTCTAGTTCC), *LUX* (CGGAGTTTTCCACCGCAAAG and TCTGACGCCATCTCTCACAG), *UCH3* (CTGATAGCCATCTCTAAGAGAACCT and CTAGGAATGCATTCTCTAAACCATGA), *PP2a* (CTTGGTGGAGCTAAGTGAAGACC and CGCCCAACGAACAAAT CACAGA), and *Tub2* (ACACCAGACATAGTAGCAGAAATCAAG and ACTCGTTGGGAGGAGGAACT).

### Intracellular protein localization assay

Protoplasts from adult leaves of Arabidopsis Col plants grown under 12 h light/12 h dark were isolated by previously described methods^46,47^. In these experiments 2 μg of the plasmid carrying 35 S::*UCH3:GFP* was used for transfection to approximately 100.000 protoplasts. After transfection the protoplasts were placed in a growth chamber with 12 h light/12 h dark with the low intensity of light. Images of UCH3:GFP localization in protoplasts was obtained by the confocal microscopy FV3000 (Olympus).

### Accession numbers

Accession numbers of genes used for constructing the phylogenetic trees for UCHs are; UCH3 [*S. lycopersicum*]: XP_004241989, UCH3-like [*S. lycopersicum*]: XP_004245809, Predicted UCH3 [*I. nil*]: XP_019190657, Hypothetical protein [*A. alpina*]: KFK24044, Predicted protein [*H. vulgare*]: BAJ98225, UCH3-1 [*O. sativa*]: XP_025878536, Predicted protein [*H. vulgare*]: BAJ95818, Unnamed protein [*T. aestivum*]: SPT19304, Predicted protein [*H. vulgare*]: BAK02661, Unnamed protein [*T. aestivum*]: SPT20700, UCH3-2 [*O. sativa*]: XP_015633691, Predicted protein [*H. vulgare*]: BAK04925, UCH2 [*O. sativa*]: XP_015626407, Hypothetical protein [*A. alpina*]: KFK25858, Predicted protein [*H. vulgare*]: BAK02146, Unnamed protein [*O. sativa*]: BAG86678, Predicted UCH2 [*I. nil*]: XP_019188703, UCH2 [*S. lycopersicum*]: XP_004246255, and Hypothetical protein [*A. alpina*]: KFK41005.

## Supplementary information

Supplementary figure 1, Supplementary figure 2, Supplementary figure 3, Full-length gel/blot images
