# Supplementary material for: Ubiquitin carboxyl-terminal hydrolases are required for period maintenance of the circadian clock at high temperature in Arabidopsis
Source: Sci Rep. 2019 Nov 19;9:17030. doi: 10.1038/s41598-019-53229-8 (PMC6863813; doi:10.1038/s41598-019-53229-8)

Supplementary Information

Ubiquitin carboxyl-terminal hydrolases are required for period maintenance of the circadian clock at high temperature in Arabidopsis

Ryosuke Hayama, Peizhen Yang, Federico Valverde, Tsuyoshi Mizoguchi, Ikuyo Furutani-Hayama, Richard D. Vierstra, and George Coupland

Supplementary figure 1

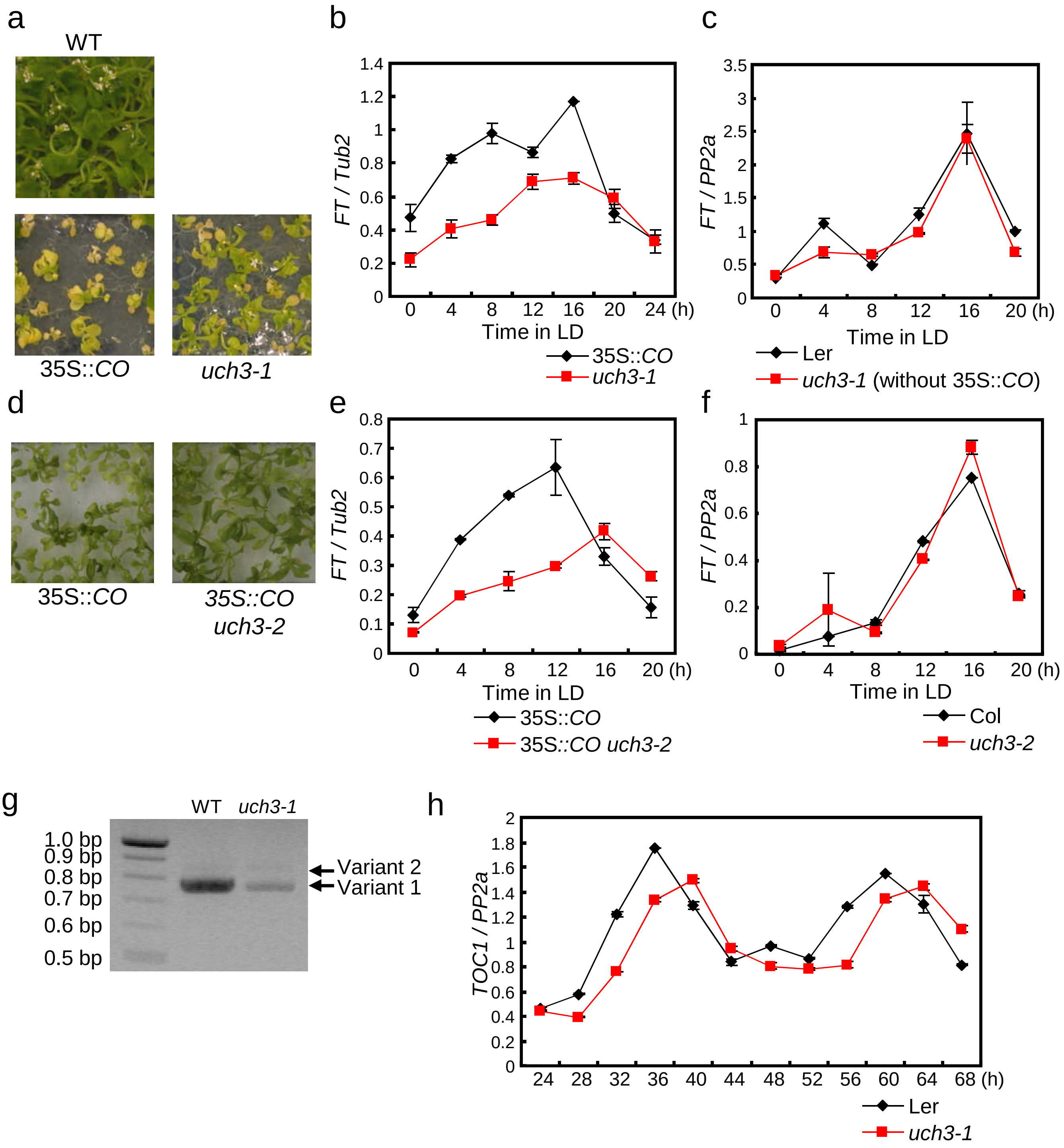

Supplementary figure 1. Characterization of *uch3-1* and *uch3-2* mutants. (a) Survival of the *uch3-1* mutant in continuous blue light. Ler, 35S::*CO* and *uch3-1* were grown in continuous blue light for two weeks, and survival of these lines was measured by monitoring levels of necrosis and chlorophyll bleaching. The 35S::*CO* line and *uch3-1* are both Ler background. **(b)** Expression of *FT* in the *uch3-1* mutant under LD. 35S::*CO* and *uch3-1* grown under 16 h light/ 8 h dark for 9 days were collected every 4 h over 24 h, and *FT* expression was monitored. *Tub2* (AT5G62690) was used for the control. **(c)** Expression of *FT* in *uch3-1* without 35S::*CO* under LD. Ler and *uch3-1* without 35S::*CO* grown under 16 h light/ 8 h dark for 12 days were collected every 4 h over 24 h, and *FT* expression was monitored. *PP2a* was used for the control. Error bars indicate SE of two biological replicates. **(d)** Survival of the *uch3-2* mutant with 35S::*CO* in continuous blue light. 35S::*CO* and *uch3-2* with 35S::*CO* were grown in continuous blue light for two weeks, and survivability of these lines were measured by monitoring levels of necrosis and chlorophyll bleaching. These lines are both Col background. 35S::*CO* in Col background used for this assay is generally slightly more resistant to blue-light exposure than that in Ler background, which represents the difference in the degree of the necrotic phenotype between the two 35S::*CO* lines in **(a)** and **(d)**. **(e)** Expression of *FT* in *uch3-2* / 35S::*CO* under LD. Experiment was carried out as indicated in **(b)**. **(f)** Expression of *FT* in *uch3-2* under LD. Experiment was carried out as show in **(c)**, except that Col was used for the control. **(g)** The agarose gel image of PCR fragments amplified from WT and *uch3-1* cDNA with primers designed at exon 1 and exon 9 of the *UCH3* locus, which exhibits major and faint bands on the WT lane and two bands on *uch3-1* lane as well. The major band in WT lane originates from mature RNA species without any introns. The two bands observed in the lane for *uch3-1* represents variant 1 and 2, which were sequenced, analyzed and shown in Fig. 1A. **(h)** Circadian expression of *TOC1* in the *uch3-1* mutant in continuous light (LL). Plants were entrained in 16 h light/ 8 h dark for 9 days and shifted to LL. Circadian expression of *TOC1* was monitored every 4 hours between time 24 and 68 after transfer to LL. Error bars indicate SE between two technical replicates.

Supplementary figure 2

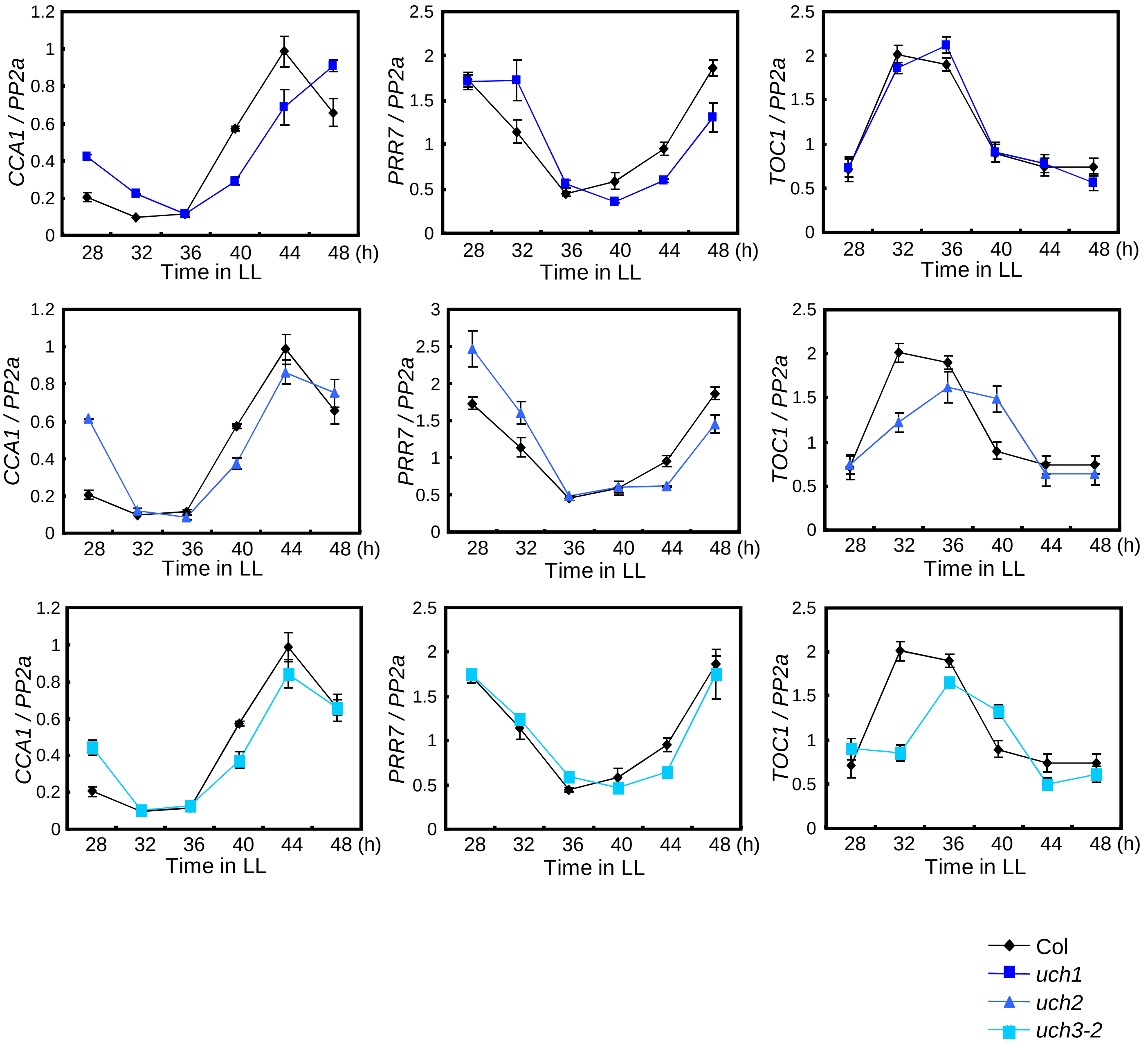

Supplementary figure 2. Effects of *uch1*, *uch2*, *uch3* and *uch1uch2uch3* mutations on clock gene expression in LL. Panels display the same results shown in Fig. 2, but gene expression in each of *uch1*, *uch2* and *uch3* mutants in a single panel in Fig. 2 was separately displayed to compare to WT alone.

Supplementary figure 3

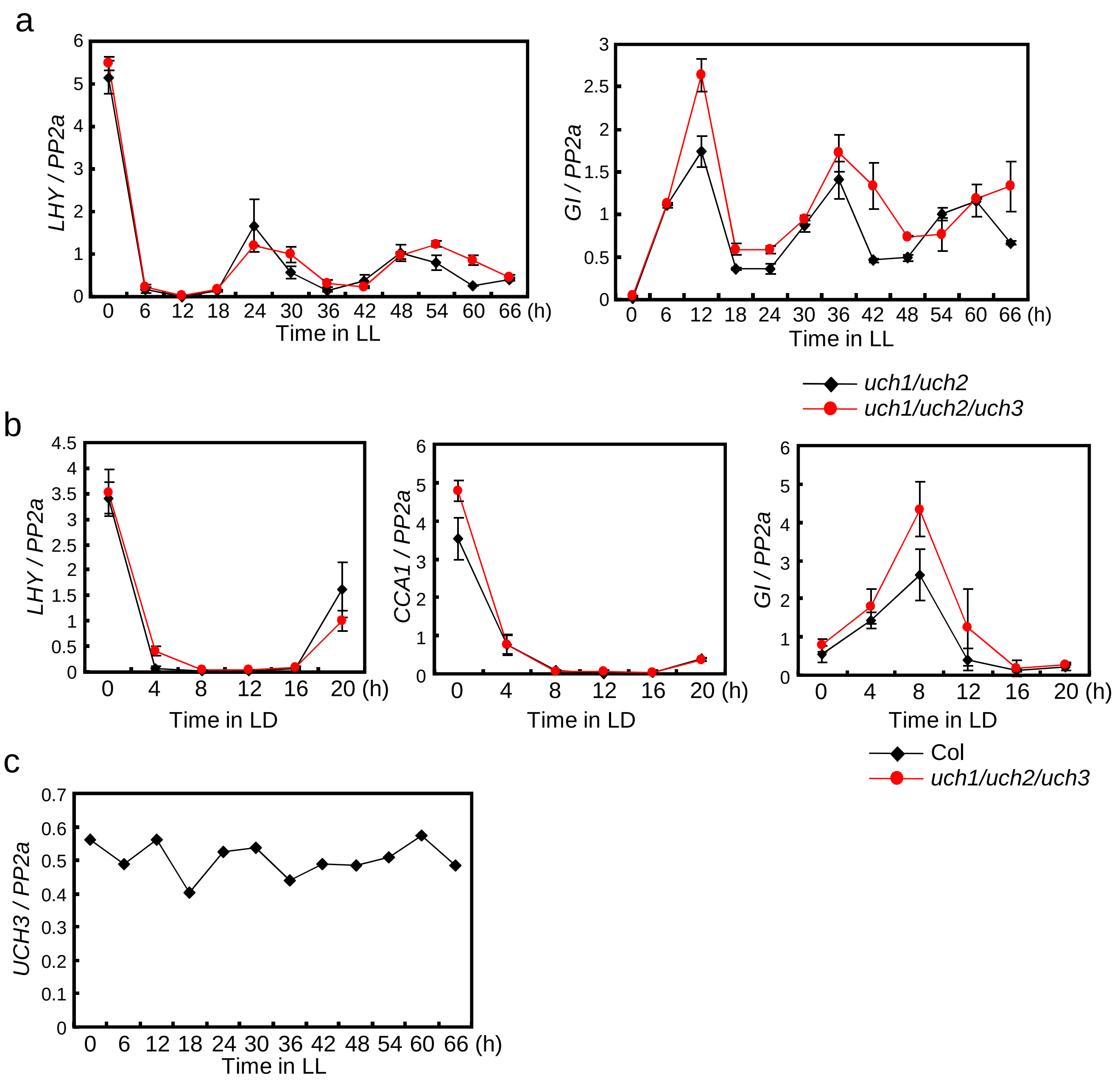

Supplementary figure 3. Circadian phenotypes of *uch* mutants in LL/LD, and *UCH3* expression in LL (a) Comparison of the circadian phenotype between *uch1 uch2* mutants and *uch1 uch2 uch3* mutants. Plants entrained to 12h light/12h dark at 22 ℃ for 12 days were sifted to LL at 29 ℃. Expression rhythms of *LHY* and *GI* were monitored every 6 h over 66 h in LL. Error bars indicate SE of two biological replicates. **(b)** The circadian phenotype in the *uch* triple mutant in light/dark cycles at a high temperature. Col and the *uch* triple mutant grown in 12h light/12h dark at 22 ℃ for 12 days were shifted to 29 ℃ at the time of the light-on, and on the second day plants were harvested every 4 h to check expression rhythms of *LHY*, *CCA1* and *GI*. Error bars indicate SE of two biological replicates. **(c)** Expression of *UCH3* transcripts at high temperature. WT RNA samples used for experiments in Fig. 6 were subjected to the expression analysis.

Full-length gel images for Fig. 2

a

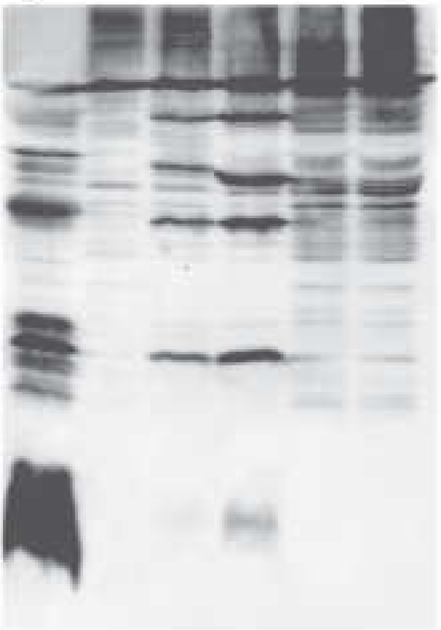

b

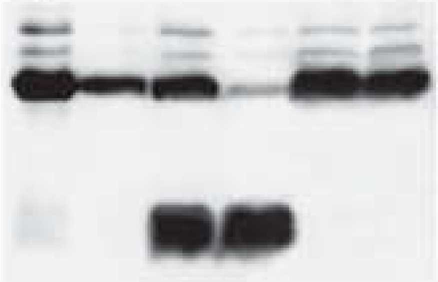

c

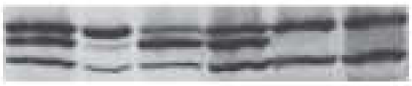

d

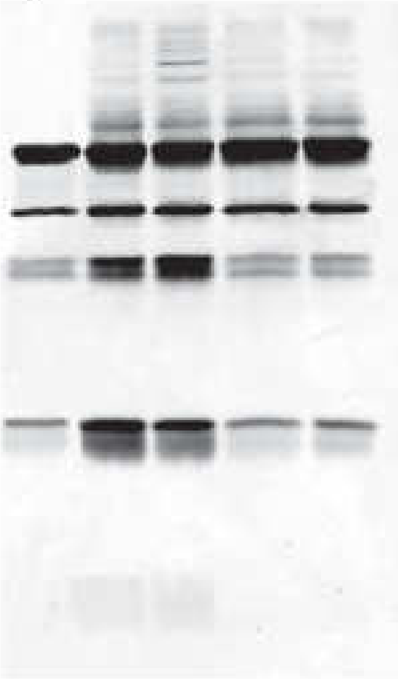

e

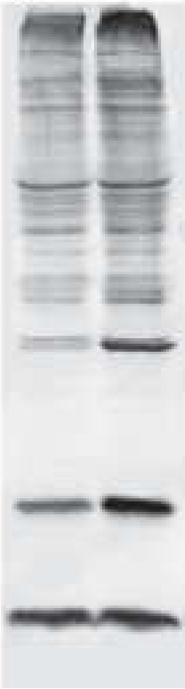

f

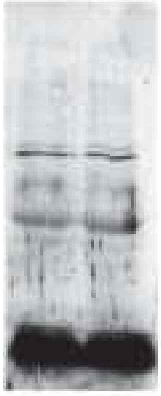

Full-length gel images for Supplementary fig. 1g

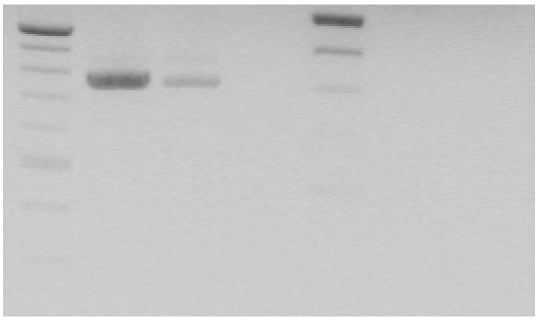

Supplement: Supplementary file 1 — Supplementary figure 1, Supplementary figure 2, Supplementary figure 3, Full-length gel/blot images [file 41598_2019_53229_MOESM1_ESM.pdf]
